# Improving recruitment to a study of telehealth management for long-term conditions in primary care: two embedded, randomised controlled trials of optimised patient information materials

**DOI:** 10.1186/s13063-015-0820-0

**Published:** 2015-07-19

**Authors:** Mei-See Man, on behalf of the Healthlines Study Group, Jo Rick, Peter Bower, on behalf of the MRC-START Group

**Affiliations:** School of Social and Community Medicine, University of Bristol, Canynge Hall, 39 Whatley Road, Bristol, BS8 2PS UK; National Institute of Health Research (NIHR) School for Primary Care Research, Manchester Academic Health Science Centre, Centre for Primary Care, the University of Manchester, Oxford Road, Manchester, M13 9PL UK; Medical Research Council North West Hub for Trials Methodology Research, Manchester Academic Health Science Centre, Centre for Primary Care, University of Manchester, Oxford Road, Manchester, M13 9PL UK

**Keywords:** Recruitment, Patient information, Research methodology, Randomised controlled trial, Primary care

## Abstract

**Background:**

Patient understanding of study information is fundamental to gaining informed consent to take part in a randomised controlled trial. In order to meet the requirements of research ethics committees, patient information materials can be long and need to communicate complex messages. There is concern that standard approaches to providing patient information may deter potential participants from taking part in trials. The Systematic Techniques for Assisting Recruitment to Trials (MRC-START) research programme aims to test interventions to improve trial recruitment. The aim of this study was to investigate the effect on recruitment of optimised patient information materials (with improved readability and ease of comprehension) compared with standard materials. The study was embedded within two primary care trials involving patients with long-term conditions.

**Methods:**

The *Healthlines* Study involves two linked trials evaluating a telehealth intervention in patients with depression (*Healthlines Depression*) or raised cardiovascular disease risk (*Healthlines CVD*). We conducted two trials of a recruitment intervention, embedded within the *Healthlines* host trials. Patients identified as potentially eligible in each of the *Healthlines* trials were randomised to receive either the original patient information materials or optimised versions of these materials. Primary outcomes were the proportion of participants randomised (*Healthlines Depression*) and the proportion expressing interest in taking part (*Healthlines CVD*).

**Results:**

In *Healthlines Depression* (n = 1364), 6.3 % of patients receiving the optimised patient information materials were randomised into the study compared to 4.0 % in those receiving standard materials (OR = 1.63, 95 % CI = 1.00 to 2.67). In *Healthlines CVD* (n = 671) 24.0 % of those receiving optimised patient information materials responded positively to the invitation to participate, compared to 21.9 % in those receiving standard materials (OR = 1.12, 95 % CI = 0.78 to 1.61).

**Conclusions:**

Evidence from these two embedded trials suggests limited benefits of optimised patient information materials on recruitment rates, which may only be apparent in some patient populations, with no effects on other outcomes. Further embedded trials are needed to provide a more precise estimate of effect, and to explore further how effects vary by trial context, intervention, and patient population.

**Trial registration:**

Current Controlled Trials: *Healthlines Depression* (ISRCTN27508731) on 26 June 2012; and *Healthlines CVD* (ISRCTN14172341) on 5 July 2012

**Electronic supplementary material:**

The online version of this article (doi:10.1186/s13063-015-0820-0) contains supplementary material, which is available to authorized users.

## Background

Randomised controlled trials are the ‘gold standard’ for evaluating health technologies, yet core aspects of trial methodology are not, in turn, underpinned by evidence. For example, recruitment is an essential part of almost all trials and yet it remains problematic, with around half of all trials failing to recruit to target and within time [1, 2]. However, little rigorous quantitative research has been conducted that can inform recruitment. A recent Cochrane review identified only 45 randomised evaluations of recruitment interventions that had been embedded in real trials (i.e. where patients in the host trial are randomised to different recruitment methods in the embedded trial). The review authors concluded that ‘trialists should include evaluations of their recruitment strategies within their trials’ (p. 22) [3].

### Increasing the evidence base for recruitment

The MRC-START research programme is a Medical Research Council (MRC) funded study, which seeks to increase the evidence base for recruitment by developing a platform to encourage the rapid and rigorous testing of recruitment interventions. This is done by embedding recruitment interventions in multiple host trials simultaneously. A fuller description of the MRC-START model and study has been provided elsewhere [4].

### Recruitment of host trials

As part of the MRC-START programme, principal investigators (PIs) of trials recently funded by the National Institute of Health Research (NIHR) Health Technology Assessment Programme or on the Primary Care Research Network portfolio were invited to participate in MRC-START. Interested trials were selected on the basis of size (see ‘Sample size’ section) and design (using a recruitment method amenable to the MRC-START interventions). Host trials were offered access to one of two interventions: optimised written patient information materials or multimedia information presented via the Internet; both intended to improve communication of trial information to potential participants. This paper reports the findings of the first two trials testing optimised printed materials.

### The host trial – The *Healthlines* Study

The *Healthlines* Study was a large primary care research programme consisting of two multicentre trials that both evaluated the potential clinical and cost-effectiveness of telephone support and computer-based self-management compared to usual care alone. The telehealth intervention was delivered by NHS Direct health information advisors, trained to provide structured support following an evidence-based conceptual model. This included regular telephone support for use of computerised packages (e.g. computerised cognitive behavioural therapy software packages) and home monitoring (e.g. blood pressure monitoring).

The telehealth intervention was used in separate trials in two patient populations: those with increased risk of cardiovascular disease (*Healthlines CVD*) and those with depression (*Healthlines Depression*) [5]. Both *Healthlines* trials identified potential participants by carrying out anonymised, code-based searches of patient records within general practices. The practice staff then mailed the patient information sheet and covering letter. Using such methods, trials in primary care settings can recruit from many practices, which is an effective method of reaching a required sample size. However, the *proportion* of patients taking part in trials often remains low, which can extend recruitment schedules, increase costs, and can raise doubts about the generalisability of research because of highly selected samples. Patient uptake in studies involving telehealth interventions may be especially problematic, with up to a 75 % refusal rate from those invited to take part in telehealth trials [6]. A recent systematic review revealed that, across eight studies, approximately one third of patients refused to take part in telehealth interventions, with the most common reasons being lack of interest and/or believing self-monitoring was not required [7]. Moreover, some patient groups are particularly difficult to recruit into research, such as those with depression [8]. An intervention which improves the proportion of invited patients choosing to participate by providing better information could have both logistical and scientific benefits, as well as potentially improving the experience of the trial for patients.

### Recruitment intervention – optimised written patient information material

Appropriate information is fundamental to providing fully informed consent. However, research ethics committee requirements mean that standard participant information can be long and complex [9, 10], which may have a negative impact on patient understanding and eventual recruitment, particularly where information sheets are visually unappealing, confusing or raise anxiety. Moreover, members of the public and members of ethics committees often have different views on what is of concern with regard to a trial and its procedures [11]. Involving consumers in the development of patient information can more closely match the information with the needs and concerns of potential participants, resulting in higher relevance and readability, without increasing anxiety [12].

In MRC-START, a process involving consumer feedback, expertise in writing for patients and graphic design is used to produce an optimised version of the patient information materials (patient information sheet and covering letter) for each host trial. Potential participants in the host trial are then randomised to receive standard or optimised patient information.

### Aims

The MRC-START project aims to assess whether improvements in the readability and presentation of patient information materials results in changes in wider outcomes, specifically the numbers responding to invitations to participate in a trial and the numbers ultimately randomised.

## Methods

The MRC-START optimised patient information materials were embedded within the *Healthlines Depression* and *Healthlines CVD* trials. The embedded recruitment intervention involved a change to trial procedures, to assess whether optimised patient information materials improved the proportion of patients responding positively to an invitation to take part in each trial and, within *Healthlines Depression* only, the proportion actually randomised.

### Development of the recruitment intervention

The process involves optimisation of readability and navigation of the documents, using expertise and considerable experience in writing for patients, as well as drawing on evidence of what works [13] and expertise in information graphic design. The revisions are then informed by repeated user testing [14–17], where the ability of patients to locate and understand key pieces of information is evaluated objectively through multiple versions. The optimised sheet covers the same topics as the original, but differs in *form* (i.e. appearance, structure and wording) and *effectiveness* (i.e. the ability to transmit the required information). For the user testing, we recruited healthy members of the public, who had a similar socio-demographic profile (age, education and occupation) to the likely samples in the *Healthlines* trials. We excluded people who had taken part in any healthcare trial or readability testing in the previous 6 months.

In the user testing, each participant saw only one version of the information [14, 15]. We conducted three rounds of user testing, with ten different participants in each round. We aimed to ensure some variation in participant characteristics in each round as well as consistency between rounds. In each round of 10 participants there were: at least 3 of each gender; no more than 2 higher education graduates; at least 4 people aged 18–39 years and at least 1 each aged in their 40s, 50s, 60s and 70–74 years. We also excluded anyone who had been treated for depression (or heart disease, as applicable) in the previous 6 months. Participants were recruited from the participant database held by Luto Research Limited (Leeds, UK), who were commissioned to undertake the user testing; participants were members of the public who had come forward for readability testing studies. They were paid a small fee to compensate them for their time.

The first round tested the original *Healthlines* materials, after which the revised versions were developed. The second and third rounds tested the revised versions, with minor changes made to wording and layout in response to the findings of each round of testing. In user testing, each participant first read a version of the patient information sheet and covering letter and was then asked to respond to 20 factual questions: 3 related to the covering letter and 17 to the information sheet. The questions were drawn from four categories of information that would apply to any trial: the nature and purpose of the trial (three questions); the process and meaning of consent (four questions); trial procedures (ten questions); safety, efficacy and nature of the tested intervention (three questions). For each question, participants were asked to locate the answer in the letter or sheet (testing navigation and organisation of the information), then give the answer in their own words (testing understanding). The data derived from user testing is indicative, not definitive, and so no particular scoring threshold was used for revisions to be required. Problems locating an answer suggest a need for clearer navigation or structure; problems in understanding suggest a need for re-wording. The decision to revise the materials is also influenced by the commentary from the person who conducted the user testing – this can indicate the severity of a problem within a document.

The original *Healthlines* patient information materials for both trials were 8-page A5 patient information sheets in booklet form, together with an A4 covering letter from the general practitioner (GP).

The patient information sheet was designed in accordance with National Research Ethics Service (NRES) guidance and based on materials used in a related earlier survey. The patient information materials were developed and revised through discussion amongst the *Healthlines* research team, and then reviewed for plain language by another researcher, external to the *Healthlines* team, with relevant expertise.

The new versions of the *Healthlines* information sheets evaluated in the embedded trials were presented as 4-page A4 booklets and were divided into 8 sections (as compared with 15 sections in the original versions), with contrasting colour and a larger font used for section headings, to aid navigation. The front page contained a ‘bulleted’ list of trial summary information, logos, a contents list and contact details, which were jointly intended to aid reader navigation and have visual appeal. The re-wording of the information sheets included greater use of lay terms, short sentences and paragraphs. Additionally, the covering letters were revised by shortening them by around one third, particularly by removing content that was replicated in the information sheets, as well as adding ‘bullets’ for lists and using bold, lower case text for emphasis. Examples of the original and revised patient information material and covering letters used in *Healthlines Depr*ession are available as Additional files 1, 2, 3 and 4. Data on word count and readability scores [18] can be found in Additional file 5. The letters were reduced in length by the process, although the patient information sheets were increased. Readability was maintained or improved in all cases. It should be noted that word length and readability indices are presented for illustrative purposes only, as they are only partial indicators of the difficulty of a document and changes in these were not the primary focus of the process.

#### Description of the *Healthlines* host trials and the embedded recruitment trials

Both embedded trials of recruitment interventions were conducted over a 4-month period (March 2013 to June 2013) towards the end of the *Healthlines* recruitment window at the final 3 participating primary care practices in the Bristol area (from a total of 43 practices involved in *Healthlines*). Practices serving populations from a range of inner city, suburban and rural settings were recruited with the help of the Primary Care Research Network (now NIHR Clinical Research Network, http://www.crn.nihr.ac.uk/). For efficiency we restricted inclusion in the embedded trial to practices with patient list sizes of 9000 or more, using computer systems compatible with the *Healthlines* search query (EMIS-LV, EMIS Web, and TPP SystmOne), and in one of three clinical commissioning groups in the South West for which the study had ethical approval. Letters of invitation, which briefly explained the nature of the study and commitment expected from each practice, were sent to practices meeting our criteria and identified by the Primary Care Research Network. Interested practices returned an expression of interest form, which was passed onto researchers to initiate relevant meetings.

Potentially eligible participants were identified from practice lists. For *Healthlines Depression*, the eligibility criteria were aged at least 18 and a confirmed diagnosis of clinical depression using standardised measures. For *Healthlines CVD,* participants were aged 40–74, with 20 % or greater risk of having a cardiovascular event in the next 10 years (QRISK2 score [19]) and had to have at least 1 of 3 modifiable risk factors (systolic blood pressure of 140 or greater; a body mass index (BMI) ≥ 30; or current smoker). Additional inclusion criteria were access to a telephone, the Internet and having an Email address for personal use. There were no additional inclusion or exclusion criteria for the embedded trials.

Standard *Healthlines* procedure was for lists to be screened by GPs, and for patients with known exclusion criteria to be removed. A researcher assisted with searches and then randomised patients to receive either the original or optimised patient information materials (using simple randomisation in a 1:1 ratio). Each practice list was randomised separately following the same procedure: each patient was assigned a computer-generated random number using Excel (Microsoft Inc., Redmond, WA, USA). The list included an ID number for each patient, rather than any identifiable information, thereby reducing the risk of any selection bias on the part of the researcher conducting the randomisation. The list was then sorted by random number, with one half assigned to the optimised patient information materials and the other half to the original.

Patients were then sent the appropriate allocated information materials as part of the invitation to participate in The *Healthlines* Study. Due to an expectation of lower recruitment, a reminder letter was mailed out to non-responders after 2 weeks in *Healthlines Depression* (an administrative error meant that all non-responders were sent the standard letter, regardless of initial allocation in the embedded trial). No reminder was used in *Healthlines CVD*. Patients were blind to the embedded recruitment trial (i.e. they were not told they were part of a recruitment trial – see ‘Research ethics approval’), while researchers knew about the trial, but were blind to patient allocation.

The patient was requested to respond by returning either a valid consent form or a decline form in a pre-paid, pre-addressed envelope. If a patient did not wish to participate, they had the option of providing reasons on the decline form. In order to carry out pre-screening, interested patients in *Healthlines Depression* were also asked to complete and return a standardised depression assessment tool, the Patient Health Questionnaire-9 (PHQ-9) [20]. If the patient returned a consent form, researchers then contacted them to ask further eligibility screening questions. For *Healthlines Depression*, those patients who scored 10 or more on the PHQ-9 were telephoned and verbally completed another standardised depression assessment, the Clinical Interview Schedule - Revised (CIS-R) [21]. Subsequently, all eligible patients were randomised on a 1:1 ratio to receive either the *Healthlines Depression* intervention and usual care, or usual care alone.

The process for *Healthlines CVD* was slightly different. Importantly, the number of patients actually randomised within each practice was restricted. This was because practice staff were required to carry out further eligibility assessments and staff availability was a concern. In particular, a CVD risk assessment was carried out by staff at the practice in order to calculate the patient’s QRISK2 score. The assessment involved taking clinical measures (e.g. blood pressure, weight and height) and collecting information that is required to compute a QRISK2 score. As covered in more detail above, patients with a 10-year CVD risk of 20 % or greater, as well as one or more modifiable risk factors (high blood pressure, overweight, or current smoker) were deemed eligible. Following further eligibility screening by the researchers, the first 25 eligible patients to complete this process at each practice were randomised on a 1:1 basis to the *Healthlines CVD* trial.

### Outcome measures

For the embedded trial of optimised patient information materials in *Healthlines Depression*, the primary outcome was the proportion of patients randomised. Secondary outcomes were the proportion of patients who accepted the offer of invitation to participate, and the proportion of eligible patients who actively opted out of the trial (i.e. returned a ‘decline’ form). Although ‘harm’ in this context could include reduced recruitment in the intervention group, we did not measure other potential ‘harms’ (such as perceptions of increased pressure to participate in the intervention group).

For the embedded trial of optimised patient information materials in *Healthlines CVD,* the primary outcome was the proportion of patients who responded positively to the invitation to participate. This, rather than actual randomisation, was selected as the primary outcome because of a cap on recruitment numbers whereby only the first 25 eligible participants were randomised in each practice. This upper limit was implemented because of practice staff availability to carry out these assessments, and an initial agreement with researchers that 25 patient assessments would be sufficient to reach target recruitment across participating GP practices. The secondary outcome in *Healthlines CVD* was the proportion of eligible patients who actively opted out (i.e. returned a ‘decline’ form).

### Sample size

The sample size calculations for the *Healthlines* host trials are detailed in the original protocol [5].

In the MRC-START programme, recruitment of host trials is guided by a generic sample size calculation which suggests a minimum of 400 patients to be approached in each arm of the host trial (note that this is the number of patients approached, not the number randomised which is the basis of standard sample size calculations). This generic calculation is detailed in the published protocol paper [4].

Since the embedded trials were planned to take place in only a subset of all the primary care practices participating in *Healthlines*, data from the host trials were already available on the numbers of potentially eligible patients identified, responding, and randomised with which to inform the sample sizes of the embedded studies. Prior to the embedded trials, approximately 500 and 250 patients per practice were sent invitations to *Healthlines Depression* and *Healthlines CVD* respectively. Of these, approximately 5 % were randomised in *Healthlines Depression*, and 25 % responded positively in *Healthlines CVD*. By conducting the embedded trials within 3 practices, absolute differences of around 6–7 % in the primary outcomes in both embedded trials would, therefore, be detectable with 80 % power and 5 % 2-sided alpha. While the corresponding relative effects, particularly in *Healthlines Depression* of a greater than 2-fold increase in randomisation rate may seem implausible for the intervention under investigation, this was a pragmatic decision based on the time it took to prepare and obtain approvals for the optimised information materials. Indeed, the original intention was to include four GP practices, but there was a delay in obtaining ethical approval to implement the optimised patient information sheets.

### Data analysis

Analyses were conducted in line with a standard statistical plan developed at Barts and the London Pragmatic Clinical Trials Unit by SE and VM (details available from the authors). Preliminary graphical and tabular examination of the data explored baseline comparability of trial arms and representativeness of the sample in terms of the overall eligible population. Outcomes were first described separately by arm, and then compared using logistic regression to estimate the between-group odds ratio (OR) and corresponding 95 % confidence interval (CI) on the basis of the intention-to-treat principle. A planned secondary analysis was performed to explore whether the impact of the intervention was moderated by gender. This was done by inserting the appropriate interaction term in the logistic regression models. All analyses were conducted using Stata version 12.1 (Stata Corp., College Station, TX, USA). The statistician conducting the analyses remained blind to allocation until all analyses were completed.

### Research ethics approval

The MRC-START programme of embedded trials of recruitment interventions was approved by the NRES Committee, Yorkshire and the Humber – South Yorkshire (Reference: 11/YH/0271) on the 5 August 2011.

The *Healthlines* host trials were approved by the NRES Committee, South West, Frenchay, Bristol (Reference: 12/SW/0009). The embedded trials of recruitment interventions were added later as a substantial amendment, with optimised versions of the patient information sheets and covering letters submitted for approval (granted 14 March 2013). As the core information for patients was unchanged, but offered in a different format, approval was given so that participants did not need to be informed that they were taking part in an embedded trial of a recruitment intervention. This also served to avoid bias and the potential of misunderstanding by patients.

### Trial registration

The *Healthlines* trials are registered with Current Controlled Trials: *Healthlines Depression* ISRCTN27508731 and *Healthlines* CVD ISRCTN14172341. The embedded trials of recruitment interventions were added as sub-studies to the original trial registrations.

## Results

### Embedded trial of recruitment interventions in Healthlines Depression

**Fig. 1:**
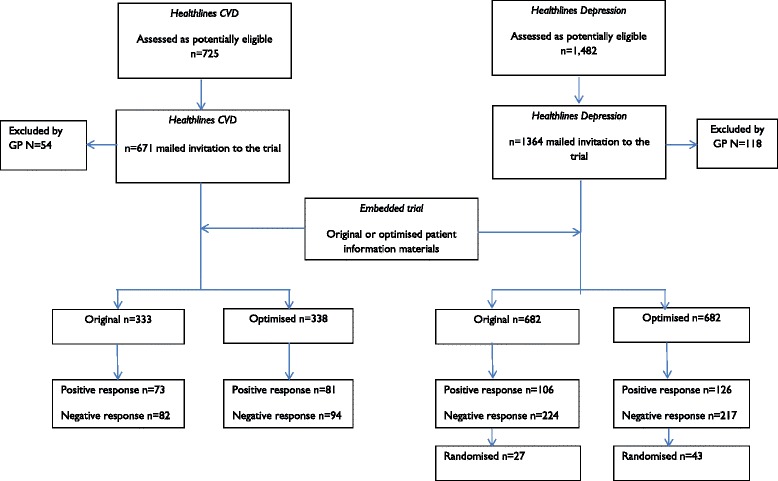
Recruitment flowchart for *Healthlines* embedded trials

A total of 1364 patients with depression were included in the analysis (all patients randomised to the embedded trial, n = 682 per group, see Fig. 1 for the recruitment flow chart). Comparison of baseline characteristics showed a similar mean age (both mean 53) and similar proportions of men (34 % in optimised materials group and 33 % in the group receiving the original materials – see Tables 1 and 2).

**Table 1:** Baseline comparability of patients in the *Healthlines Depression* Study

Patient characteristic	Optimised		Standard	
	Number	Percent	Number	Percent
Total	682	100.0 %	682	100.0 %
Gender				
Male	230	33.7 %	228	33.4 %
Female	452	66.3 %	454	66.6 %
Mean age in years (SD)	52.5	(17.2)	52.7	(17.5)

**Table 2:** Baseline comparability of patients in the *Healthlines CVD* Study

Patient characteristic	Optimised		Standard	
	Number	Percent	Number	Percent
Total	338	50.4 %	333	49.6 %
Gender				
Male	245	50.4 %	241	49.6 %
Female	93	50.3 %	92	49.8 %
Mean age in years (SD)	66.3	(5.6)	66.7	(5.2)

The proportion randomised in the *Healthlines Depression* trial was 43/682 (6.3 %) among those receiving optimised patient information materials and 27/682 (4.0 %) in the standard patient information group (OR = 1.63, 95 % CI = 1.00 to 2.67, *p* = 0.05, Table 3).

**Table 3:** Number and proportion of patients randomised to *Healthlines Depression* by recruitment intervention

Randomised to *Healthlines Depression*	Patient information		Total
	Standard	Optimised	
	Number (%)	Number (%)	Number (%)
No	655 (96)	639 (94)	1294 (95)
Yes	27 (4)	43 (6)	70 (5)
Total	682 (100)	682 (100)	1364 (100)

OR = 1.63, 95 % CI = 1.00 to 2.67, *p* = 0.05

The proportions responding positively to the invitation to participate in *Healthlines Depression* were 126/682 (18.5 %) among those receiving optimised patient information materials compared to 106/682 (15.5 %) in the standard patient information group (OR = 1.13, 95 % CI = 0.93 to 1.64, *p* = 0.15). The proportions who actively declined to participate in *Healthlines Depression* were 217/682 (31.8 %) among those receiving optimised patient information materials compared to 224/682 (32.8 %) in the standard patient information group (OR = 0.95, 95 % CI = 0.76 to 1.20, *p* = 0.69). There was no evidence that the optimised patient information materials were differentially effective at achieving randomisation among men and women (Table 4).

**Table 4:** Number and proportion of patients randomised to *Healthlines Depression* by recruitment intervention and gender

Gender	Randomised to *Healthlines Depression*	Patient information		Total	Odds ratio
		Standard	Optimised		
		Number (%)	Number (%)	Number (%)	
Male	No	220 (96.5)	215 (93.5)	435 (95)	
	Yes	8 (3.5)	15 (6.5)	23 (5)	1.919
*Sub-total*		*228*	*230*	*458 (100)*	
Female	No	435 (95.8)	424 (93.8)	859 (95)	
	Yes	19 (4.2)	28 (6.2)	47 (5)	1.512
*Sub-total*		*454*	*452*	*906 (100)*	
Total		682 (50)	681 (50)	1363 (100)	

Interaction OR = 0.79, 95 % CI = 0.27 to 2.28, *p* = 0.660

### Embedded trial of recruitment interventions in Healthlines CVD

A total of 671 potential participants were included in the analysis (all participants randomised to embedded trial, n = 338 in the optimised group, 333 in the original – see Fig. 1 for the recruitment flow chart). Comparison of baseline characteristics showed a similar mean age (both mean 67) and similar proportions of men (72.5 % in optimised materials group and 72.4 % in the group receiving the original materials).

The proportion responding positively to the invitation to participate in *Healthlines CVD* was 81/338 (24.0 %) among those receiving optimised patient information materials and 73/333 (21.9 %) in the standard patient information group (OR = 1.12, 95 % CI = 0.78 to 1.61, *p* = 0.53, Table 5).

**Table 5:** Number and proportion of patients accepting the invitation to participate in *Healthlines CVD* by recruitment intervention

Responded positively to *Healthlines CVD* invite	Patient information		Total
	Standard	Optimised	
	Number (%)	Number (%)	Number (%)
No	260 (78)	257 (76)	517 (77)
Yes	73 (22)	81 (24)	154 (23)
Total	333 (100)	338 (100)	671 (100)

OR = 1.12, 95 % CI = 0.78 to 1.61, *p* = 0.53

The proportions who actively declined the invitation to participate in *Healthlines CVD* were 94/338 (27.8 %) among those receiving optimised patient information materials compared to 82/333 (24.6 %) in the standard patient information group (OR = 1.18, 95 % CI = 0.84 to 1.66, *p* = 0.35). There was no evidence that the optimised patient information materials were differentially effective at encouraging a positive response among men and women (Table 6).

**Table 6:** Number and proportion of patients accepting the invitation to participate in *Healthlines CVD* by recruitment intervention and gender

Gender	Accepted invite to *Healthlines CVD*	Patient information		Total	Odds ratio
		Standard	Optimised		
		Number (%)	Number (%)	Number (%)	
Male	No	183 (75.9)	188 (76.7)	371 (76.3)	
	Yes	58 (24.1)	57 (23.3)	115 (23.7)	0.957
*Sub-total*		*241*	*245*	*486 (100)*	
Female	No	77 (83.7)	69 (74.2)	146 (78.9)	
	Yes	15 (16.3)	24 (25.8)	39 (21.1)	1.786
*Sub-total*		*92*	*93*	*185 (100)*	
Total		333 (100)	338 (100)	671 (100)	

Interaction OR = 1.87, 95 % CI = 0.81 to 4.30, *p* = 0.143

## Discussion

We tested the effects of optimised patient information materials on recruitment into two host trials evaluating a telehealth self-management intervention. In a group of patients with depression, the overall rates of participation were low, but receiving the optimised patient information materials did result in a small increase in the proportion randomised. However, the absolute numbers recruited were small and the lower confidence limit around the estimate was consistent with no effect. There was no evidence of effect on patients at risk of cardiovascular disease, nor of effects on other outcomes in either population.

### Limitations

The number of patients invited to participate in *Healthlines Depression* exceeded our generic sample size requirements for the embedded trial, but the numbers approached for *Healthlines CVD* were lower.

Aligning the work of the host and embedded recruitment trials was identified as important in qualitative work undertaken to develop the MRC-START programme [22]. However, it is often difficult to achieve this in practice, especially where recruitment studies are not embedded from the beginning of the host trial. The time required to prepare the optimised materials (approximately 6 weeks) meant that the embedded trials could only be conducted in 3 practices, rather than the 4 originally planned. Where possible, embedded trials would be linked to hosts far earlier in the process, preferably during the bid development, or around the time of the funding decision. Compromises may be required in cases where alignment is not possible, but this may be justified given the very limited evidence base for recruitment at present.

The difficulties of aligning embedded and host trials was illustrated by an error in study procedures. All participants were successfully randomised to receive standard or optimised information initially. However, the reminder letter (used only in the *Healthlines Depression* trial) was not randomised due to an administrative error, and all participants in *Healthlines Depression* received the standard reminder letter. Our expectation would be that this would potentially dilute the impact of the intervention, although it is theoretically possible that the change in materials may have been salient to participants and more likely to evoke a response.

The preferred outcome measure for MRC-START interventions is the number randomised to the host trial. We were unable to test for an effect on the randomisation rate in *Healthlines CVD*. This was due to the cap placed on recruitment in the host trial, necessary for operational reasons. Whilst real-world trials provide the best context for examining the effectiveness of interventions aimed at improving recruitment, there will inevitably be occasions when decisions about the management of the host trial can place limitations on the design and delivery of the embedded trial.

Our intervention used a defined and published process to optimise the content and presentation of patient information sheets. It is important to note that many researchers routinely involve patients in the development of their information materials, and the impact of the optimised materials tested here may be lessened where patients have already had significant involvement, or where the trial team have significant prior expertise. Our work with other trials has highlighted significant variation in this regard. However, it is important to reiterate that the optimisation process tested here is not the same as conventional patient involvement in the development of trial materials. This is especially true in the degree to which the materials are formally *tested* for their ability to impart information.

We assessed a single moderator variable (gender) as part of our pre-specified analytic plan. Host trials differ markedly in terms of the baseline characteristics recorded, and gender is one variable which will be present in most data sets and coded similarly. Our use of gender as a moderator reflects these pragmatic considerations, rather than being based on a strong theoretical rationale about the impact of gender on the effects of enhanced patient information. Other variables (such as socio-economic status or health literacy) may be more relevant but are less likely to be consistently recorded.

### Interpretation of the findings in the context of the wider literature

There is limited literature on the effects of modified information on trial recruitment. In the previously mentioned Cochrane review, three studies explored the impact of supplementary written material on recruitment and found little evidence of benefit. No previous studies have looked at optimised participant information materials of the type evaluated in this study [3].

When compared on the same outcome (positive response to an invitation to participate in *Healthlines*) there was no effect of the optimised PISs in either patient population. Due to constraints in the host trials, it was not possible to test effects on rates of randomisation across both trials, and it is not clear if the modest positive impact on proportions of patients randomised is particular to depressed patients. Although patients with depression have been found to hold positive attitudes to research participation [23], recruitment to depression trials is notoriously difficult [24, 25]. The effects of depression on concentration, attention and motivation have been associated with difficulties using written materials [26]. It is possible these challenges mean that depressed patients are more able to benefit from the optimisation process, and thus explain the larger intervention effect in this group.

It should also be noted that improvements in patient randomisation rates are not the only outcome of optimised materials, which may also impact on improved understanding, enhanced shared decision-making, and better patient experience. These outcomes are not routinely measured in trials. They could be tested through bespoke measures, but this would add a significant logistical overhead to trials, and our discussions have often indicated that trials are unwilling to add additional measures. Alternatively, the addition of nested qualitative work as part of a programme of mixed methods research might be a suitable approach to further develop our understanding of the wider impact of optimised patient materials.

### Implications for recruitment practice

If it is assumed that the effect seen in *Healthlines Depression* is robust, the small increase in the rate of recruitment is likely to have fairly limited advantages in terms of the external validity of the study sample. However, the demonstrated increase in the rate of recruitment could have significant logistical benefits for a larger definitive trial, in terms of the number of practices that would need to be recruited, or the number of invitation letters to be sent. For example, at a 4 % recruitment rate, recruiting 600 patients to a definitive trial would require 15,000 invitations, while a recruitment rate of 6 % requires only 10,000 invitations. Obviously these potential advantages need to be set against the financial and opportunity costs of the optimisation process (approximately £7000 at current rates), but such calculations are an important part of study planning, and relatively small benefits in recruitment rates may be attractive to trial teams in the context of large studies. These logistical benefits must also be viewed in the context of other potential benefits that are more difficult to quantify, such as improved understanding and patient satisfaction.

### Generalisability

Our study provides evidence about the effects of optimised patient information materials on trial recruitment in the context of a telehealth intervention in two patient populations. Further trials are needed to assess the effect of optimised patient information materials, to provide a more precise estimate of effect on recruitment, consider effects on retention and explore whether effects vary by trial context, intervention and patient population. The MRC-START team plan to conduct analyses of the effects of two recruitment interventions (either optimised patient information materials or a multimedia decision aid, by comparison with standard information) across twelve host trials.

## Conclusions

Evidence from these two embedded trials suggests limited benefits of optimised patient information materials on randomisation rates, which may only occur in particular patient populations. Further embedded trials of these materials are being conducted to both improve the precision of these findings, and explore how their effects may vary by different factors: trial context (e.g. primary care or other settings), intervention (e.g. different types of optimisation), and patient populations (including demographic and clinical characteristics). Findings from this research will be included in the Cochrane systematic review of interventions to improve recruitment to trials [27]. A more comprehensive cohort of embedded trials of recruitment interventions across the trials portfolio could lead to a rapid development of the evidence base around recruitment, to make trials more acceptable and accessible to patients.

## Additional files

Additional file 1:
**Original version of the covering letter for the** ***Healthlines Depression*** **trial.**
Additional file 2:
**Optimised version of the covering letter for the** ***Healthlines Depression*** **embedded trial.**
Additional file 3:
**Sample page from the original version of the participant information booklet (** ***Healthlines Depression*** **trial).**
Additional file 4:
**Sample page from the optimised version of the participant information booklet (** ***Healthlines Depression*** **embedded trial).**
Additional file 5:
**Healthlines participant information material word counts and SMOG index readability scores.**

## Abbreviations

BMI: body mass index
CIS-R: Clinical Interview Schedule - Revised
CVD: cardiovascular disease
GP: general practitioner
MRC: Medical Research Council
MRC-START: Systematic Techniques for Assisting Recruitment to Trials
NIHR: National Institute of Health Research
NRES: National Research Ethics Service
OR: odds ratio
PI: principal investigator
PHQ-9: Patient Health Questionnaire-9
QRISK2: cardiovascular disease risk algorithm
