# Supplementary material for: Improving recruitment to a study of telehealth management for long-term conditions in primary care: two embedded, randomised controlled trials of optimised patient information materials
Source: Trials. 2015 Jul 19;16:309. doi: 10.1186/s13063-015-0820-0 (PMC4506607; doi:10.1186/s13063-015-0820-0)
Supplement: Additional file 1: — Original version of the covering letter for the Healthlines Depression trial. [file 13063_2015_820_MOESM1_ESM.docx]

PRACTICE LETTERHEAD

«Title» «Forename_s» «Surname»


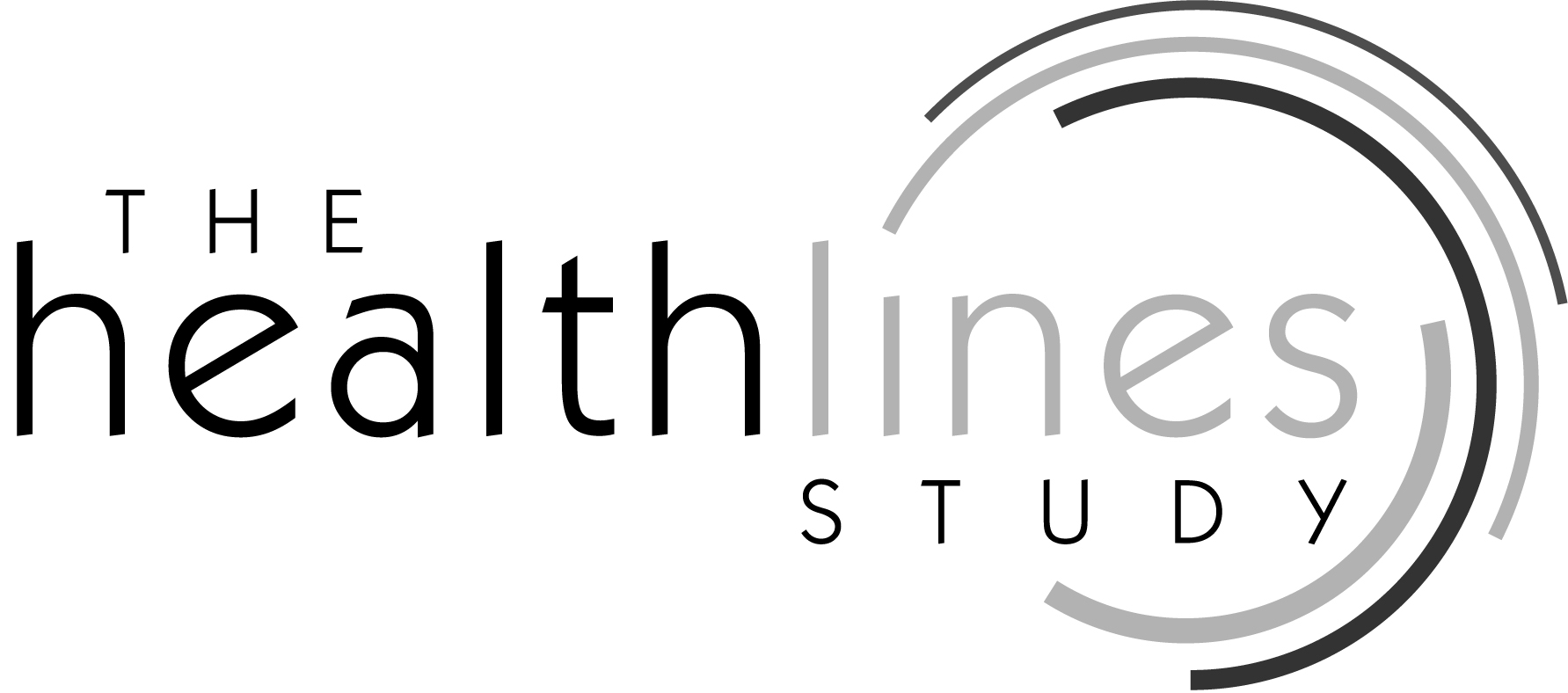


«House_nameFlat»

«No_and_street»

«Village»

«Town»

«Postcode» «Practice StudyID»/«RecruitID»

Dear «Title» «Surname», <Date>

**Researching Different Ways of Providing Support for Health and Well-being**

I would like to invite you to take part in a research study. Our practice is supporting this research, which aims to explore different ways that the NHS could help people to improve and look after their own health.

NHS Direct is planning to offer a new programme, called *NHS Direct Healthlines Service*, in which they offer extra advice, information, and support for your health through regular phone calls and the Internet. Participants will be given access to a wide range of resources on the Internet, while those with high blood pressure will also be loaned a compact blood pressure monitor for a year. This research will explore whether this approach improves peoples’ health, is popular with users, and is cost-effective.

We have chosen a number of people who are registered at the practice, and we are inviting them to consider taking part in this study. If you decide to take part, you will be allocated by chance to one of two groups. You might continue to receive support with your health from your practice just like you do now. We will call this ‘Usual Care.’ Alternatively, you might receive Usual Care plus extra support provided by *NHS Direct Healthlines Service.* We want to find out which one of these approaches is best.

The enclosed leaflet gives you more information about the research, which is being conducted by the Universities of Bristol and Sheffield, along with NHS Direct.

**If you are interested in taking part in this research study**, please complete the *Acceptance Form* AND the *Initial Screening Questionnaire* and return them both to the research team in the freepost envelope provided. Completing these forms does not commit you to taking part. Once received, a researcher will be in touch with you to discuss the next steps and to complete a further assessment to check your eligibility for the study. This check may show that you are not eligible, in which case you will not be able to join the study, and you will be told the reason(s).

**If you do not want to participate in the study**, it would be greatly appreciated if you would complete the anonymous *Decline Form* and return this to the research team in the freepost envelope. Not taking part will not affect the care you receive from your GP in any way.

If you have any questions about the research, please contact xxxxxxxx xxxxxx at the University of Bristol by telephone (xxxx xxxxxxx) or email (xxxxxxxx xxxxxxxx). Thank you for your time and consideration.

Yours sincerely,
