# Supplementary material for: Improving recruitment to a study of telehealth management for long-term conditions in primary care: two embedded, randomised controlled trials of optimised patient information materials
Source: Trials. 2015 Jul 19;16:309. doi: 10.1186/s13063-015-0820-0 (PMC4506607; doi:10.1186/s13063-015-0820-0)
Supplement: Additional file 2: — Optimised version of the covering letter for the Healthlines Depression embedded trial. [file 13063_2015_820_MOESM2_ESM.doc]

PRACTICE LETTERHEAD

«Title» «Forename_s» «Surname»


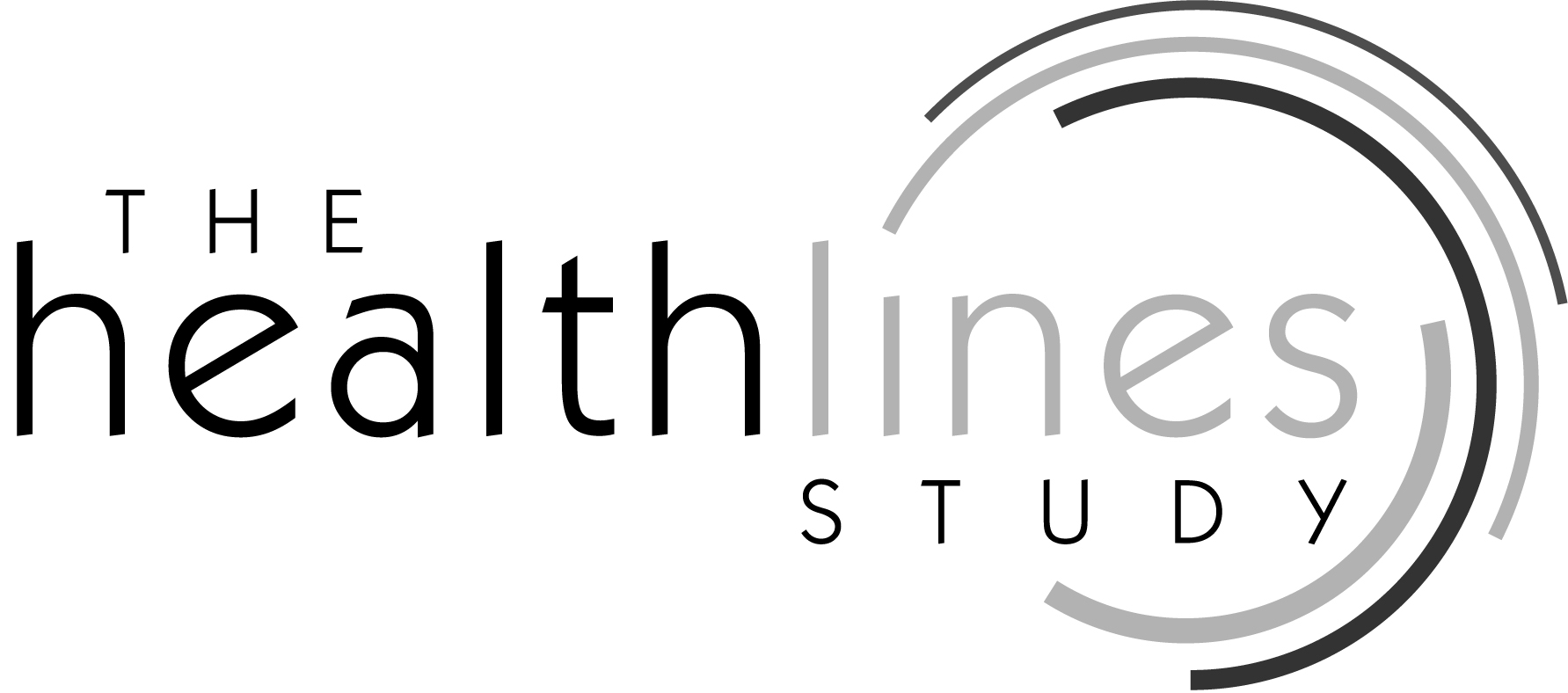


«House_nameFlat»

«No_and_street»

«Village»

«Town»

«Postcode»

«Pracitce StudyID»/«RecruitID»

<Date>

Dear «Title» «Surname»,

**An invitation to take part in a study of different ways to support people’s health**

The NHS is looking at new ways to support patients, including by phone and the internet. The NHS Direct Healthlines Service is a new service that will provide advice, information and support, in addition to that provided by your GP.

This new service is being tested in a study and our practice is supporting this research. The study is being run by the Universities of Bristol and Sheffield, and NHS Direct.

We have selected some patients who have recently visited the GP because of feeling down, stressed or worried. We have sent this letter to you, to ask if you would be willing to take part.

The study will assess what patients think about the NHS Direct Healthlines Service, what effect it has on their health and on other NHS services they use. The enclosed booklet gives details about the study – please feel free to discuss it with others.

**If you would like to take part, please:**

- fill in the ‘Acceptance form’ and the ‘Initial Screening Questionnaire’, and
- send both to the University in the envelope provided (no stamp needed).

Returning the ‘Acceptance form’ does not commit you to taking part.

**If you would prefer not to take part, please:**

- fill in the ‘Decline form’, and
- send it to the University in the envelope provided (no stamp needed).

Returning the ‘Decline form’ does not reveal your name to us. Saying no to the study will not affect the care you receive from your GP.

If you have any questions, please contact ........ at the University of Bristol on (..........) or email (.........). Thank you for taking time to read about the study.

Yours sincerely,
