# Supplementary material for: Improving recruitment to a study of telehealth management for long-term conditions in primary care: two embedded, randomised controlled trials of optimised patient information materials
Source: Trials. 2015 Jul 19;16:309. doi: 10.1186/s13063-015-0820-0 (PMC4506607; doi:10.1186/s13063-015-0820-0)
Supplement: Additional file 3: — Sample page from the original version of the participant information booklet ( Healthlines Depression trial). [file 13063_2015_820_MOESM3_ESM.doc]

**What will taking part involve?**

If you are eligible to participate in the study, you will first complete a study consent form, and then a questionnaire. This will ask about your health and lifestyle behaviours (e.g., diet, physical activity), any difficulties you have in getting healthcare, satisfaction with the care you receive, and how you are managing your health. You will have the option of completing this online or requesting a copy by post, and it should take about one hour in total.

Next, you will be allocated by chance to one of two groups. This is done completely randomly by a computer, so that everyone has an equal opportunity of being in one group or the other. We do not know which way is best for improving health and well-being, but we will use the results from this study to find out.

One group will continue to receive support with their health by making appointments to see a GP or nurse at their usual general practice when this is needed. We will call this ‘Usual Care.’ The other group will also receive Usual Care, plus extra support provided by NHS Direct. We call this ‘NHS Direct Healthlines.’ This will include support provided over the telephone and Internet, including email, web-based tools, information, and online programs. NHS Direct advisors will be in regular telephone contact with you, and they will work with you to set health and lifestyle goals according to your preference and health needs. You will also be given a number of possible options for managing your health depending on your needs.

At 4, 8, and 12 months after starting the study, we will ask you to complete a questionnaire similar to the initial one (see above). At the end of the study, the research team will collect information about your care from your medical records.

A small number of people will be invited to evaluate NHS Direct Healthlines in a face-to-face interview about 6 months into the study. If you are selected for this, we will send you more detailed information nearing the interview date.

**Are there any disadvantages in taking part?**

You cannot choose which type of help you are offered. This will be determined by chance. Participating in research does mean giving up some of your time, which may not appeal to everyone. You may feel uncomfortable answering some questions over the phone. However, the depression screening questions are from a standard questionnaire, which is designed to collect information about your symptoms, rather than about your personal circumstances. Collecting this information is necessary in order for you to participate. If this is too distressing, you can either ask us to phone you on another occasion or withdraw from the study. We do not anticipate any other risks associated with taking part in this study.

**What are the possible benefits of taking part?**

It is important that we understand the views of patients in order to create services that are most likely to be accessible and helpful, as well as cost-effective. Your participation in this trial will be very helpful in planning future services to be delivered by the NHS, which may benefit future patients. You may personally benefit from taking part in this study by learning more about your health, how to manage it, and having regularly scheduled health checks. As a result, your health and well-being might improve.

**What happens when the study stops?**

After the study is completed, we hope that your health and well-being will have improved. If you still feel you need further help, your GP will continue to support and help you with this.
