# Supplementary material for: Improving recruitment to a study of telehealth management for long-term conditions in primary care: two embedded, randomised controlled trials of optimised patient information materials
Source: Trials. 2015 Jul 19;16:309. doi: 10.1186/s13063-015-0820-0 (PMC4506607; doi:10.1186/s13063-015-0820-0)
Supplement: Additional file 4: — Sample page from the optimised version of the participant information booklet ( Healthlines Depression embedded trial). [file 13063_2015_820_MOESM4_ESM.pdf]

4 What will I need to do if I take part?

Assessment

If you send back the 'Acceptance form', one of the research team will then phone you to ask some extra questions. This would take no more than 30 minutes.

This is because not everyone we write to will be suitable - the study is focussing on people who have low mood.

If you are suitable for the study, we would then ask you to fill in a consent form and answer some questions about your health and lifestyle, and how you manage your health. There are also questions about any difficulty you have in getting healthcare, and how satisfied you are with the care you receive. This questionnaire takes up to an hour to fill in and you do it at home - we can post it to you, or you can do it on your computer.

Phone and email

We also need to check that you have a phone (it could be a landline or mobile), as well as being a regular user of the internet. You would need an email address - if necessary, we can help you to arrange this.

Once it is clear that you are suitable to take part, it will be decided by chance if you will receive the NHS Direct Healthlines Service (A opposite), or your usual GP care (B opposite).

Follow-up

Whether or not you are receiving the NHS Direct Healthlines Service, we would ask you to complete questionnaires at 4, 8, and 12 months after starting the study. These would be similar to the questionnaire completed at the start of the study.

At the end of the study, the research team would collect information from your medical records, looking at any appointments you have had at the practice. Once the study is over, you would continue with usual GP care as before.

Among those patients receiving the NHS Direct Healthlines Service, we will invite a small number for a face-to-face individual interview about halfway through the study. If selected, we will send you more information nearer the time.

5 Possible benefits and disadvantages of taking part

Your taking part in this study will help in the planning of services in the NHS. This will benefit future patients.

You may benefit personally from taking part, by learning more about your health, how to manage it, and having regular health checks. As a result, your health and well-being may improve.

Taking part will mean giving up some of your time. You may feel uncomfortable answering some of the assessment questions. However, they are taken from a questionnaire used often to assess patients for depression. The questions are intended to find out about your thoughts and feelings, and not your personal life. We cannot think of any other disadvantages of taking part in the study.

6 More information about taking part

Your GP

If you take part, we will notify your GP. We will also send them a summary of the assessment we make at the start of the study and the follow-up questionnaires you complete. If you are also getting the NHS Direct Healthlines Service in the study, we will tell your GP about your health and well-being.

Who is running and funding the study?

The research is being run by the Centre for Academic Primary Care at the University of Bristol, as well as the Universities of Sheffield and Southampton, and NHS Direct. It is funded by the NHS National Institute for Health Research (NIHR).

All research in the NHS is looked at by a Research Ethics Committee, to protect your safety, rights, well-being and dignity. The Healthlines Study has been reviewed and approved by the South West (Frenchay) NHS Research Ethics Committee.

The study is due to finish in 2014. The results will be written up in a report to the NHS and published for use by health professionals and NHS managers. All patients who take part will be sent a summary of the results. We will also send you a free copy of the full report if you request one.
