# Supplementary material for: Improving recruitment to a study of telehealth management for long-term conditions in primary care: two embedded, randomised controlled trials of optimised patient information materials
Source: Trials. 2015 Jul 19;16:309. doi: 10.1186/s13063-015-0820-0 (PMC4506607; doi:10.1186/s13063-015-0820-0)
Supplement: Additional file 5: — Healthlines participant information material word counts and SMOG index readability scores. [file 13063_2015_820_MOESM5_ESM.docx]

**Additional file 5 Healthlines word counts and SMOG index readability data**

**Original versions**

CVD trial letter 407 (14.9)

Depression trial letter 447 (16.3)

CVD trial PIS 2,162 (16.1)

Depression trial PIS 2,049 (16.5)

**START versions**

CVD trial letter 315 (14.9)

Depression trial letter 305 (15.0)

CVD trial PIS 2,283 (14.7)

Depression trial PIS 2,196 (14.9)

For the SMOG index, a lower score indicates more readable / less difficult text.

Online SMOG calculator used: http://www.niace.org.uk/current-work/readability
